# Determinants of Lack of Recovery from Dependency and Walking Ability Six Months after Hip Fracture in a Population of People Aged 65 Years and Over

**DOI:** 10.3390/jcm11154467

**Published:** 2022-07-31

**Authors:** Enrique González Marcos, Enrique González García, Josefa González-Santos, Jerónimo J. González-Bernal, Adoración del Pilar Martín-Rodríguez, Mirian Santamaría-Peláez

**Affiliations:** 1 RACA 11 Artillery Regiment, Cid Campeador Military Base, 09193 Burgos, Spain; enriquegojs@gmail.com; 2 Traumatology and Orthopedic Surgery Department, Burgos University Hospital (HUBU), 09006 Burgos, Spain; egonzalezga@saludcastillayleon.es (E.G.G.); amartinro@saludcastillayleon.es (A.d.P.M.-R.); 3 Department of Health Sciences, University of Burgos, 09001 Burgos, Spain; jejavier@ubu.es (J.J.G.-B.); mspelaez@ubu.es (M.S.-P.)

**Keywords:** hip fracture, age, aging, recovery of independence, recovery of walking ability

## Abstract

Background: Hip fracture in the elderly means that between a quarter and a half of patients do not regain the levels of independence and walking ability that they previously had, according to the literature, after the fracture. Material and methods: Retrospective study of 537 patients aged ≥65 years who survived at the sixth month after fracturing their hip, of which the age, sex, type of fracture, surgical risk, independence (BI), walking ability, cognitive level (PS), comorbidities, indicated drugs, complications, surgical delay, hospital stay, and surgical technique are known. Using Pearson’s χ^2^ test, all the variables were contrasted with respect to the limitation or not, at the sixth month of the recovery of both independence and pre-admission walking ability. Multivariate analysis provides the necessary adjustment to the previous contrast. Results: We have found that age and PS ≥ 5 at admission limit recovery from both dependency and walking ability. Surgical risk, independence (BI) upon admission, anemia, and constipation during the hospital stay limit the recovery of the BI. Worsening of walking ability during the hospital stay and the type of extra-articular fracture, which was surgically treated by osteosynthesis, limit the recovery of walking ability. Conclusions: The factors previously exposed, and perhaps the fact that patients with hip fractures are not routinely referred to rehabilitation, explain the high proportion of patients who do not recover their previous independency (36%) or walking ability (45%) to the fact of fracturing.

## 1. Introduction

Hip fracture is the second most common fragility fracture after wrist fracture [1]. Between 28% and 35% of people aged ≥65 years have at least one fall at the same height per year that can potentially end in a fracture, and this incidence increases with age. It is called “multi-fall syndrome”, which affects 30–50% of the institutionalized elderly population [2]. The incidence of hip fracture in Spain was 2.1% each year between 1997 and 2010, a year in which it was 325 cases in men and 766 in women for every 105 inhabitants, and it affects more significantly those aged 85 years or older [3].

The most pessimistic information about the percentage of elderly people who recover their previous function after suffering a hip fracture is 23% [4], but the most optimistic estimate that it can reach more than half, in which case the functional deficit baseline, 25-hydroxy-vitamin D deficiency and complication with “delirium” [5,6] are the most limiting factors for mobility recovery.

Among the instruments used to standardize the measurement of the physical health of the elderly related to the activities of daily living (ADL), the Barthel Index (BI) [7] in its Spanish version [8] has been chosen as it is widely used in geriatrics.

In previous studies, there are multiple scales used to assess mobility and gait. The Tinetti scale [9] is one of them, the “Cumulated Ambulation Score” (CAS), described by Foss N.B., et al. in 2006 [10] and used by Danish authors [11]. Other scales are gait-specific, such as the aforementioned FIM scale [12], which has a module that assesses gait function. The “Functional Ambulation Classification” (FAC) was also initially described more than three decades ago for the evaluation of walking ability in stroke patients [13,14], but it has been used in elderly patients with hip fractures, too, is used in this studio [15,16], and has the advantage of its simplicity in clinical application. It is the one that we will apply in a summarized way, as it is exposed in material and methods.

Much research studies the recovery of patients in the context of rehabilitation programs. However, the results are not conclusive and more research is required [17]. However, there are not so many who study the factors that may limit the recovery of these patients.

A greater fear of falling after a hip fracture is related to the female sex, polypharmacy, poor physical functioning and daily activities, and depressive symptoms one year after the fracture occurred [18]. Frölich et al., in a prospective cohort study, found that those who were the frailest patients were the ones who failed to return to their independent living, but they consider that the majority of the community-dwelling patients returned to independent living only with a minor increase in care needs; they also consider that standing within 24 h from hip fracture surgery was vital in maximizing short-term functional recovery [19]. One systematic review proposed the hand grip strength and frailty as emerging significant predictors of poor functional outcomes and mortality in the literature, in addition to other predictors grouped in medical factors (comorbidity, anesthesia, sarcopenia), surgical factors (delay in intervention, type of fracture), socio-economic factors (age, sex, ethnicity) and system factors including lower case-volume centers [20]. Age, male sex, trochanteric fracture, preoperative delay, postoperative drainage use, serum albumin, and ADL at discharge and internal fixation are related to functional recovery [21,22]. Some of these factors can also influence mortality after hip fracture as advanced age, male sex, living in a rural area, diabetes, tumor, preoperative delay, and postoperative drainage use [22].

This research aims to study which factors exist in our population of patients aged ≥65 years, which limit, and to what degree, the recovery of the situation of independence (BI), as well as their ability to walk prior to suffering a hip fracture.

The hypothesis of this study is based on the fact that the factors that denote poor basal functioning, as well as the presence of health problems and other complications, will be factors that may influence the recovery of the baseline situation.

## 2. Materials and Methods

### 2.1. Study Design—Participants

In a retrospective longitudinal study, all patients were treated at the University Hospital of Burgos (HUBU). Inclusion criteria: Patients aged 65 years or older who, by a low energy mechanism, suffered a hip fracture in the biennium 14 March 2019–14 March 2021. All patients admitted to the HUBU with these characteristics were included in the study, followed after discharge from the outpatient clinics of the Orthopedic Surgery and Traumatology Service of the same hospital through face-to-face and non-face-to-face consultations through interviews with the patients, their families, and/or responsible caregivers. Exclusion criteria: Patients with peri-prosthetic fractures, peri-synthesis fractures, and pathological fractures, that is, on bones affected by primary tumor or metastasis, were excluded from the study; likewise, patients who were referred to other hospitals without completing the treatment or follow-up period for any cause, except death. Data collection was carried out on all patients who were admitted to the emergency room for hip fractures and underwent surgery by the Orthopedic Surgery and Traumatology Service.

### 2.2. Sample Size

The sample size was estimated following the procedure for finite populations, using the formula $n=\frac{N\times {\left(Z_{\alpha }=1.96\right)}^{2}\times p\times q}{\delta^{2}\times \left(N-1\right)+{\left(1.96\right)}^{2}\times p\times q}$. The known population reported by the National Institute of Statistics (INE) (https://www.ine.es/jaxiT3/Tabla.htm?t=2852, accessed on 25 May 2022) and a similar study [23] was taken into account, establishing a proportion of hip fractures in the population of 0.389% (*p* = 0.000398, and its complementary *q* = 0.99602) and assuming a sampling error of 1% (*δ*^2^ = 0.01). Based on this, it was concluded that the sample should be made up of 152 patients with hip fractures under care by the HUBU.

### 2.3. Main Outcomes—Instruments

The head of the Traumatology Section of the Orthopedic Surgery and Traumatology (OST) Service was responsible for collecting the data from each participant’s electronic medical record for further analysis. In order to study variables that may influence cognitive impairment, sociodemographic data such as age (dichotomized in <85 and ≥85 years) and sex (woman/man) and clinical data such as the type of fracture (intracapsular/extracapsular), the type of treatment (surgical/conservative), the surgical technique (arthroplasty/synthesis), complications during admission such as “delirium” or constipation, the surgical risk assessed according to the American Society of Anesthesiologists Physical Status Classification (ASA) [24], prescription of different drugs before admission and after hospital discharge, and concomitant pathologies at the time of admission. The main variable refers to ambulation capacity according to the functional ambulation classification (FAC) [10,11] (categorized their levels 4–5 as “good”, 3–4 as “regular”, and 0–1 as “bad” walking ability).

There are multiple ways to standardize the measurement of the physical health of the elderly: the activities of daily living (ADL) index (“Activities of Daily Living” or “ADL”) [7] and the instrumental activities of daily living (IADL) [8]. In specific questions of mobility, the functional independence measure (FIM) [9] is available, which is fundamentally validated for patients with neurological diseases, and its application is complex. The Barthel Index (BI) [10] in its Spanish version [11] has been chosen because it is the most widely used tool in the functional assessment of elderly patients suffering from hip fracture [12,13,14,15,16]. The categorization of the BI has been performed in four: “1” (BI = 100): fully independent, “2” (100 < BI ≥ 90): slightly dependent, “3” (90 < BI ≥ 60): moderately dependent and “4” (BI < 60): severely or totally dependent. “BI Recovery” is the difference between the BI (variable with four categories 1 to 4) at the income and at the sixth month, so that, if the value is negative, it is understood that they did not recover. “Walking ability recovery” is the category difference in “walking ability” at admission and at the sixth month so that “they do not recover” if said difference is a negative value. The cognitive impairment was assessed using Pfeiffer Scale (PS) [25]. It is a questionnaire that collects the number of errors of the evaluated patient when ten simple questions are posed and establishes four categories of the definition of cognitive impairment depending on the dependence of people in the intellectual area: 0–2 errors is the absence of deterioration or autonomy in the intellectual area, 3–4 errors is slight impairment and help of other people in intellectually complex matters, 5–7 errors is moderate deterioration and require help on a regular basis but not always, and 8–10 errors denote severe deterioration and continuous supervision. In the present study, cognitive impairment according to PS is expressed as a dichotomous variable: absence of cognitive impairment or mild impairment (PS ≤ 4 errors) and moderate or severe cognitive impairment (PS ≥ 5 errors). Data on FAC, BI, PS, and institutionalization prior to admission, at discharge, and at 6 months if the patient survives is collected. All clinical or sociodemographic information is obtained in the emergency department, on the hospitalization floor, or in face-to-face or telematic consultations after hospital discharge.

### 2.4. Statistical Analysis

To characterize the sample, the mean and standard deviation (SD) were used in the case of continuous variables and absolute frequencies and percentages if the variables were categorical. Both categorical variables from more than two categories and continuous variables were dichotomized based on previous studies and tended to obtain groups as homogeneous as possible. Bivariate analyses were performed to study the relationship between clinical features at “BI Recovery” and “Walking ability recovery”, 6 months using the Pearson independence test (χ^2^), as well as the likelihood ratio. In the analyses with significant results, the ratio of advantages or “odds” (OR) with its limits (lower/upper) was also obtained. In addition, in order to quantify the magnitude of relationships of bivariate analysis and identify possible predictive factors of main variables at 6 months, depending on the different clinical characteristics, an analysis was performed using binary logistic regression, where dichotomous dependent variables are “BI Recovery” and “Walking ability recovery”. All the significant variables obtained in the previous bivariate analysis were included as independent in the referred multivariate study, and the OR = e^βi∗(±Δi)^ with its limits (lower/upper) was also obtained too.

Statistical analysis was performed with SPSS software version 25 (IBM-Inc., Chicago, IL, USA). For the analysis of statistical significance, a *p*-value < 0.05 was established.

## 3. Results

### 3.1. Recovery of the Initial Situation

**Figure 1:**
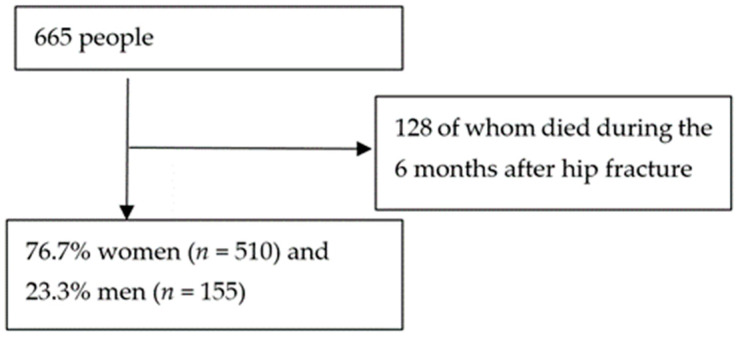
Participants flow.

The study sample consisted of 665 people, 128 of whom died during the 6 months after hip fracture. The age of the participants was between 65 and 102 years, with a mean of 86.2 years, 76.7% women (*n* = 510) and 23.3% men (*n* = 155) (Figure 1). In the group of surviving patients in the series, 36.1% did not regain independence at the sixth month, nor did 44% regain walking ability prior to the fracture.

### 3.2. Influence on Lack of Recovery by 6th Month of the Category of the BI Prior to Admission

#### 3.2.1. Regarding the Previous Situation or Admission

In the bivariate analysis carried out between the BI recovery variable (yes/no) with the variables studied that take into account the situation before the patient was admitted (Table 1), an association was found with age ≥ 85 years, type of extracapsular fracture, also comorbidities such as chronic renal failure and high blood pressure, likewise the use of antihypertensive drugs, all of which are risk factors for non-recovery. There is also a relationship with independence (BI at admission ≥ 60, BI at admission ≥ 90), better cognitive status (PS at admission ≤ 4), and better gait (FAC category ≤ 2). No association was found with age, sex, or institutionalization prior to admission.

Binary logistic regression (Table 2) (Nagerkelke’s R^2^ = 0.289) finds (in bold) age in completed years, surgical risk (ASA), independence (highest BI: 0–100), and cognitive impairment (number of errors in the EP) as risk factors for non-recovery of BI.

#### 3.2.2. Regarding the Effect That the Fracture and Admission Exert

We obtained the bivariate analysis (Table 3), and the following risk factors were found: the use of synthesis as a surgical technique, hospital stay ≥ 11 days, BI at discharge < 90, deterioration of at least one category in the BI between hospital admission and discharge, impairment of at least one category in the ability to walk, better cognitive status at discharge (PE ≤ 4), cognitive impairment in at least one category in the PS, “de novo” institutionalization at hospital discharge it associates greater risk than staying at home, and the following events: hemoglobinemia ≤ 8.5 mg/dL, being transfused with ≥3 packed red blood cells, delirium, and constipation. The variable’s type of treatment and surgical delay were not significant in this bivariate analysis.

The multivariate analysis (Table 4) has estimated significant variables with adjustment for not regaining independence: older age, higher BI (0–100) at hospital discharge, but above all, deterioration of BI during admission, in addition to hemoglobinemia ≤ 8.5 mg/dL, and constipation.

### 3.3. Influence on Lack of Recovery by 6th Month of the Category of the Walking Ability Prior to Admission

#### 3.3.1. Regarding the Previous Situation or Admission

Using Pearson’s χ^2^ tests and likelihood ratio (χ^RV 2^) with the dependent variable recovering (yes/no) walking ability (Table 5), risk factors have been found for non-recovery at the sixth month, age ≥ 85 years, extracapsular type of fracture, ASA III or IV surgical risk, BI < 90 prior to admission, moderate or severe cognitive impairment (PS ≥ 5), institutionalization prior to admission, comorbidities at admission: chronic anemia, heart failure, having the patient prescribed anticoagulants and proton-pump inhibitors before the fracture. The poor ability to walk before admission has been significant as a protective factor for non-recovery at six months; this effect does not change and gains greater associative strength in the multivariate adjustment. There is no association with sex or with BI at admission < 60 points.

The binary logistic regression (Table 6) has obtained the only significant results (R^2^ = 0.500) for not recovering walking ability, thanks to the adjustment, in addition to older age, the extracapsular type of fracture, surgical risk, number of errors in PS, and use of proton-pump inhibitor, on or prior to admission. IB (0–100) independence and, above all, worse ambulation (FAC) prior to admission have been protective factors for the lack of recovery of gait; these two effects are found in the bivariate analysis.

#### 3.3.2. Regarding the Effect That the Fracture and Admission Exert

In the bivariate analysis with variables during admission and at the end of it (Table 7), we have found that the following are risk factors for non-recovery of gait: surgical technique by synthesis, start of standing, and gait beyond the third postoperative day, BI < 90 at discharge, BI < 60 at discharge, poor walking ability at hospital discharge, impairment during admission of at least one category in ambulatory ability, cognitive impairment according to PS ≥ 5, loss during admission of at least one category according to the same PS, and new institution at discharge.

We have also found various complications that occurred during admission as risk factors: hemoglobinemia ≤ 8.5 mg/dL, being transfused and if performed with three or more packed red blood cells, delirium, constipation, impaired kidney function during admission, urinary tract infection (UTI), acute urine retention (AUR), need to a new prescription of vitamin D at discharge.

There are four variables: deep venous thrombosis (DVT), acute ischemic stroke (AIS) during admission, liquid thickeners, and new neuroleptics prescription at hospital discharge, which are risk factors in the analysis, but the result must be interpreted with reservation, because in the 2 × 2 table, at least one box has expected values less than 5, and therefore, despite their significance, we will not include them in the multivariate analysis.

Neither the type of treatment nor the hospital stay, nor the surgical delay influence the non-recovery of walking capacity.

Institutionalization as a residential destination (new and not new) at discharge is a protector factor in the non-recovery of walking, contrary to new institutionalization, so patients with an institutional destination at discharge have significantly greater possibilities to maintain their previous level of capacity for ambulation.

The multivariate analysis (Table 8), with binary logistic regression (R^2^ = 0.275), only confirms as true factors associated with not recovering the ability to walk the loss of at least one category of ability to walk during admission and synthesis as a technique surgery used.

**Figure 2:**
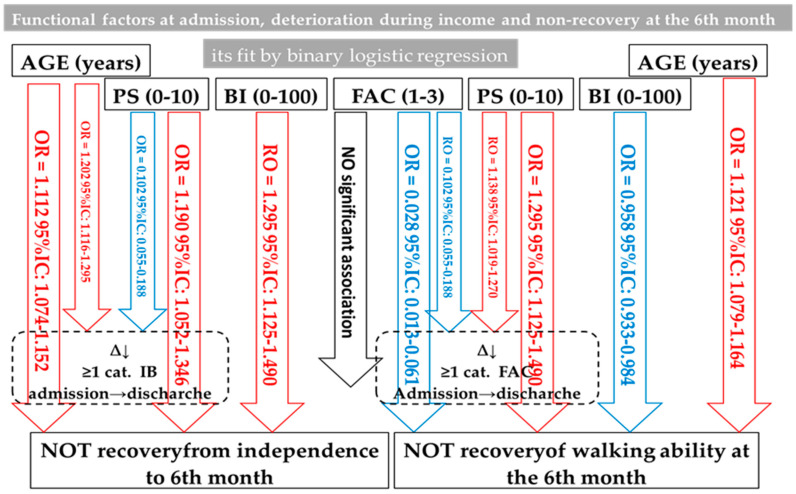
Summary of the interaction between functional factors in the elderly with hip fracture in our patients.

Functional loss during admission (see Table 9 and Table 10, as well as Figure 2) after hip fracture in the elderly in our series is basically related to cognitive impairment before said admission, but in a different direction. There is a direct relationship or risk factor regarding the deterioration of the ability to walk. On the other hand, there is an indirect relationship so that patients with greater cognitive impairment at admission experience less loss of independence during admission.

Below (Figure 2), the relationships between functional variables are exposed so that in blue, we have those that prevent and in red, those that are risk factors for non-functional recovery in the sixth month.

## 4. Discussion

Age is the factor that, in almost any publication, is associated with the limitation in the recovery of the previous function after a hip fracture in the elderly and in any period of time: 2 and 6 months [26]; 4 months [27], 6 months [28,29], 8 months [30], 1 year [31,32], 6 y 18 months [33], or not specifying a certain time, but when a more or less specific rehabilitation program ends [34,35,36,37,38,39,40,41,42]. In general, men have the worst evolution, according to much of the literature consulted [36,37,43]. According to Sylliaas et al. [44], women have a worse evolution, although there are also authors who, coincidentally with our work, do not appreciate differences [32,45]. In our study, as in the literature consulted, age, both in bivariate and multivariate analysis, is a risk factor for the non-recovery of independence and, also, for the non-recovery of ambulatory capacity.

In our investigation, the average stay is not associated with a lack of functional recovery. Martin-Martin et al. [40] associate it with worse mobility and Orive et al. [33] with BI impairment. The surgical delay in this work does not condition the functional evolution either, but there are studies in which surgical delay ≥ 48 h limits mobility [28] or the recovery of independence [33].

The pathology associated with the patient who is admitted to be treated for a hip fracture has different importance. The frailty of the elderly can be defined by the number of severe or terminal chronic diseases that the patient has [46], obtaining an index that is adjusted for age and baseline functional status. Kua J. et al. [47] have highlighted that the previously known geriatric scale [48] called Reported Edmonton Frail Scale, has a high prognostic value in all hospital admissions for acute processes in the elderly, and specifically a significant impairment (OR = 6.19, *p* = 0.01) of basic activities of daily living (ADL) [49,50] in the sixth month after hip fracture.

The number of concurrent comorbidities has been described as a factor of poor functional prognosis at four months [41] that we have not found. In fact, in our multivariate adjustment, no comorbidity influences the recovery of function at six months. Parkinson’s disease has a proven relationship with ambulatory capacity in patients with hip fractures [51]. In addition, it has been described that hypertension and diabetes are comorbidities associated with a greater limitation of functional recovery [36,41]. In addition, it has been described that hypertension and diabetes are comorbidities associated with greater limitation of functional recovery [52,53]. The greater surgical risk of our patients limits the recovery of [43] independence in terms of the BI value, not as well as the recovery of the march in our research, as other authors refer [30,33].

Several authors [51,54] associate the need for help to walk or not being able to walk alone outside the residential setting before admission with not regaining independence (IADL) [55] a year after the fracture. McGilton et al. [56] consider that poor global functional status, gait, and cognitive status at admission are limiting to recovery. Lower BI and more errors in the PS impair both the global functional status and the ability to walk Mariconda M. et al. [57] at one year. In our series, cognitive impairment prior to admission limits the recovery of both independence and gait in the sixth month after the hip fracture. The most independent patients, according to the BI before the fracture in this series, are the ones with the most limited global functional recovery (BI) in the sixth month. This phenomenon and with the same index is described in the literature [33] with prospective research at 6 and 18 months. However, in our patients, functional deterioration during admission is directly related to said previous cognitive deterioration only in the case of walking. Patients with a worse baseline cognitive situation acquire a lesser loss of their independence between admission and discharge. Similarly, patients with worse gait have at admission (higher value of the FAC variable), as occurs with dependency, with less functional reserve at admission, less loss generated by the fracture, and they maintain levels at the sixth month not as different from the previous ones. Therefore, the high value of the FAC variable prevents the non-recovery of the gait function. The essential factor so that these functions, independence and ability to walk, are not recovered is their qualitative loss during admission, especially in the case of loss of dependency (OR = 25.43, 95% CI: 12.61–51.28). Our work coincides with Dubljanin-Raspopović E. et al. [27] in that cognitive impairment is a pre-eminent factor in global functional (BI) and gait non-recovery.

Our patients from a nursing home before fracture have, after adjusting variables, a recovery of BI and gait not significantly different from those who lived at home, coinciding with Ariza-Vega P. et al. [31]. Other works instead [32,42] consider that institutionalization prior to admission limits gait recovery.

The extra-articular fracture type has, in general, a worse functional prognosis in the literature [32,40,45,52], just as we have clearly found in our multivariate analysis regarding the non-recovery of gait function. The worse prognosis in the evolution of BI can, at least in part, be explained by age since our patients with extra-articular fractures have a higher mean age, as in almost all the literature [16,53]. Di Monaco [58] does not find differences in prognosis between the types of fracture. A meta-analysis [59] showed that the use of total arthroplasty in patients with displaced intracapsular fractures gives better functional results than osteosynthesis, and total hip arthroplasty, according to prospective studies, is preferable in this type of fracture both due to its functional outcome as having fewer complications [60,61,62]. The synthesis, in our research, by the bivariate analysis, is followed by less recovery of both dependency (BI) and walking capacity at six months. This effect, in the multivariate analysis, is annulled in terms of non-recovery of BI; and persists as a risk factor in the non-recovery of walking. The mean age of our survivors does not differ significantly between those who underwent synthesis or arthroplasty. The only complications that we have been able to relate to the functional prognosis after multivariate adjustment have been anemia, coinciding with Foss N.B. et al. [10], and constipation; however, for other authors [63], they are ulcers by pressure and “delirium”.

It is a relative limitation that the measurement of the evolution at six months is a shorter time than that of some publications, which was already mentioned that they take 12 or 18 months, although there is no lack of medium-term studies: six months like ours, even at two, and four months in some cases. It has been pointed out that most of the recovery of global independence (BI) occurs in the first trimester [51]. The scientific evidence of a retrospective observational study is less than that of a cohort study, fundamentally because it is a mere consultation of registered data, no matter how rigorous the anamnesis and record of it have been. Our hip fractures do not follow any rehabilitation program, which may be related to the high percentages of lack of functional recovery that we have; in agreement with Orive et al. [33] when they state that not referring to rehabilitation increases the possibility of deterioration of the BI prior to six months, more than two times (OR = 2.34, 95% CI: 1.31–4.16) and at 18 months more than three (OR = 3.18, 95% CI: 1.62–6.25) with respect to undergoing rehabilitation treatment.

As strengths, it should be noted that the sample is large enough. Includes all fractures treated by our hospital in relation to its health area. This minimizes potential selection biases that often accompany a retrospective study. Take all possible variables. In addition to performing statistical analysis comparing dichotomous qualitative variables, binary logistic regression, in which we also incorporate quantitative independent variables for adjustment, allows us to eliminate biases such as effect modification or interaction, especially in relation to age. Although retrospective, it is still a longitudinal study, which to a large extent allows its conclusions to be taken as a valid explanation of the knowledge of the factors that truly influence limiting functional recovery in the elderly with hip fractures in our environment.

The results of this research show the factors in our population of patients aged ≥65 years, which limit, and to what extent, the recovery of the situation of independence (IB), as well as their ability to walk before suffering a hip fracture, as established in the objective of the research.

## 5. Conclusions

The factors associated with both the lower recovery of the BI and the ability to walk are older age and worse cognitive status at admission. Perhaps the lack of referral to rehabilitation of our patients is a very important factor to take into account in the poor recovery from dependency and walking.

Limitations to the recovery of independence are one’s own independence (high BI) on admission and discharge, the loss of it during admission, and the high surgical risk (ASA).

Both dependency (low BI) as well as impaired ambulatory capacity during admission limit recovery of gait.

Patients suffering from extracapsular fractures and surgical treatment by synthesis limit the recovery of walking in the sixth month. Likewise, patients taking proton-pump inhibitors prior to admission have less recovery from walking.

Hemoglobinemia < 8.5 mg/dL, as well as constipation, are the complications that are associated with a worse prognosis of dependence, but not “delirium”.

Sex does not influence, neither have any comorbidity been found, nor the greater number of concomitant comorbid processes with hip fracture related to functional prognosis

## Figures and Tables

**Table 1:** Bivariate analysis recovery of the BI (yes/no); significant factors prior to or at admission.

	No BI Recovery at 6th Month				
*Bivariate Analysis Recovery of the BI*				RO Limits	
*Prior to/at Admission*	χ^2^	*p*	RO	Lower	Upper
≥85 years	34.05	<0.001	3.255	2.183	4.854
Extracapsular hip fracture	5.05	0.025	1.511	1.052	2.172
Chronic renal insufficiency prior admission	7.63	0.006	1.891	1.220	2.931
Arterial hypertension prior admission	5.03	0.025	1.543	1.071	2.224
Antihypertensive drugs prescribed before entering	4.38	0.036	1.495	1.041	2.147
BI at admission ≥ 60	45.24	<0.001	1.716	1.589	1.853
BI at admission ≥ 90	18.05	<0.001	2.293	1.570	3.350
PS at admission ≤ 4	10.19	0.001	2.148	1.355	3.404
(FAC category ≤ 2) not bad gait (good or regular)	23.81	<0.001	16.000	3.846	66.563

BI: Barthel Index; FAC: functional ambulation classification; RO: Odds Ratio; PS: Pfeiffer Scale.

**Table 2:** Binary logistic regression of recovery of the BI (yes/no); situation prior to admission.

Prior to/at Admission	No BI Recovery at 6th Month					
R^2^ = 0.294	Coef. β	χ^2^ Wald	*p* Value	RO	L. Inf	L. Sup
Age (years)	0.107	36.506	<0.001	**1.113**	1.075	1.153
Sex male	−0.247	0.909	0.340	0.781	0.470	1.298
Extracapsular fracture	0.338	2.468	0.116	1.402	0.920	2.137
ASA III ó IV	0.478	4.923	0.026	**1.612**	1.057	2.459
BI at admission (0 a 100)	0.070	34.216	0.000	**1.073**	1.048	1.098
Walking ability (FAC categories 1–3)	−1.448	3.234	0.072	0.235	0.048	1.139
N° errors PS (0–10)	0.186	8.929	0.003	**1.204**	1.066	1.360
Arterial hypertension (yes)	0.149	0.488	0.485	1.161	0.764	1.765
Chronic renal insufficiency (yes)	0.345	1.771	0.183	1.412	0.849	2.347

BI: Barthel Index; ASA: American Society of Anesthesiologists Physical Status Classification; FAC: functional ambulation classification; PS: Pfeiffer Scale.

**Table 3:** Bivariate analysis recovery of the BI (yes/no); significant variables—effect of the fracture and outcome.

*Bivariate Analysis Recovery of the BI (Yes/No)*				RO Limits	
Events during Admission	χ^2^	*p*	RO	Lower	Upper
Synthesis as a surgical technique	5.82	0.016	1.608	1.108	2.335
Hospital stay ≥ 11 days	10.12	0.001	1.907	1.293	2.812
BI at discharge < 90	11.27	0.001	1.948	1.330	2.853
Deterioration ≥ 1 category in the BI	109.22	<0.001	14.365	8.014	25.751
Impairment ≥ 1 category (FAC 1–3) in the ability to walk	17.58	<0.001	2.192	1.526	3.147
Better cognitive status at discharge (PS ≤ 4)	7.62	0.006	1.911	1.222	2.989
Cognitive impairment in at least one category in the PS	13.79	<0.001	7.102	2.323	21.716
“De novo” institutionalization at hospital discharge	23.52	<0.001	3.262	2.014	5.282
Still remain at home when discharged from hospital	9.28	0.002	1.765	1.236	2.520
Hemoglobinemia ≤ 8.5 mg/dL	15.29	<0.001	2.278	1.515	3.425
Constipation	17.29	<0.001	2.165	1.512	3.099

BI: Barthel Index; FAC: functional ambulation classification; PS: Pfeiffer Scale.

**Table 4:** Binary logistic regression of recovery of the BI (yes/no); situation during admission.

Income Events and Effects	No Recovery of Baseline Independence					
R^2^ = 0.503	β	χ^2^ Wald	*p*	RO	L. Inf	L. Sup
Age (years)	0.115	31.046	0.000	**1.122**	1.078	1.169
Sex male	−0.120	0.161	0.688	0.887	0.495	1.592
Surgical thecnique syntesis	0.403	2.532	0.112	1.497	0.911	2.460
Hospital stay ≥ 11 days	0.507	3.622	0.057	1.661	0.985	2.801
IB (0–100) at discharge	0.025	7.886	0.0050	**1.025**	1.008	1.043
Loss of independence (BI) at least 1 category	3.236	81.737	0.000	**25.430**	12.609	51.287
Cognitive status: PS (number of errors)	0.065	0.726	0.394	1.067	0.919	1.240
Cognitive impairment at least 1 category	0.860	1.280	0.258	2.363	0.533	10.480
Walking ability at least 1 category	0.325	1.716	0.190	1.384	0.851	2.252
Institutionalization at discharge	−0.479	1.974	0.160	0.619	0.317	1.208
New institutionalization at discharge	0.320	0.638	0.424	1.378	0.628	3.022
Heglobinemia ≤ 8.5 mg/dL	0.677	4.067	0.044	**1.969**	1.019	3.803
Be transfused during admission	−0.219	0.482	0.487	0.803	0.433	1.491
“Delirium” during admission	0.388	1.665	0.197	1.474	0.818	2.657
Constipación pertinacious during admission	0.706	6.693	0.010	**2.026**	1.187	3.459

BI: Barthel Index; PS: Pfeiffer Scale.

**Table 5:** Bivariate analysis recovery of the FAC (yes/no); variables significantly associated; situation prior to admission.

*Bivariate Analysis Recovery of the Walking Ability (Yes/No)*				RO Limits	
*Prior to/at Admission*	χ^2^	p	RO	Lower	Upper
≥85 years	53.60	<0.001	4.105	2.796	6.026
Type extracapsular of fracture	7.58	<0.001	1.657	1.168	2.351
ASA III or IV surgical risk	11.60	0.001	1.853	1.309	2.623
BI < 90 prior to admission	13.60	<0.001	1.957	1.379	2.778
Cognitive impariment PS ≥ 5	8.08	0.004	1.834	1.222	2.754
Institutionalization prior to admission	11.69	0.001	1.985	1.350	2.917
Chronic anemia	4.75	0.029	1.727	1.080	2.761
Heart failure	3.64	0.057	1.513	1.009	2.269
Anticoagulant drugs prescribed before admission	3.76	0.052	1.552	1.017	2.367
Proton-pump inhibitor before admission	4.22	0.04	1.587	1.042	2.419
Bad walking ability (FAC category 3)	43.23	<0.001	0.508	0.466	0.555

ASA: American Society of Anesthesiologists Physical Status Classification; BI: Barthel Index; PS: Pfeiffer Scale; FAC: functional ambulation classification.

**Table 6:** Binary logistic regression of recovery of the FAC (yes/no); situation prior to admission.

Prior to/at Admission	No Recovery of Baseline Walking Ability at 6th Month					
R^2^ = 0.500	Coef. β	χ^2^ Wald	*p* Vaule	RO	L. Inf	L. Sup
Age (years)	0.114	34.341	0.000	**1.121**	1.079	1.164
Sex male	−0.394	1.922	0.166	0.674	0.386	1.177
Extracapsular fracture	0.537	5.236	0.022	**1.710**	1.080	2.709
ASA III ó IV	0.404	2.559	0.110	**1.498**	0.913	2.457
BI prior to admission (0 a 100)	−0.043	9.946	0.002	**0.958**	0.933	0.984
Cognitive impairment prior to admission	0.258	13.024	0.000	**1.295**	1.125	1.490
Walking ability prior to admission	−3.584	79.328	0.000	**0.028**	0.013	0.061
Institutional origin prior to admission	0.574	3.403	0.065	1.776	0.965	3.269
Chronic anemia prior to admission	0.430	1.667	0.197	1.537	0.801	2.949
Chronic renal insufficiency prior to admission	0.187	0.366	0.545	1.206	0.658	2.210
Anticoagulant drugs prior admission	−0.046	0.021	0.885	0.955	0.516	1.769
Proton-pump inhibitor prior admission	0.591	4.230	0.040	**1.806**	1.028	3.172

ASA: American Society of Anesthesiologists Physical Status Classification; BI: Barthel Index.

**Table 7:** Bivariate analysis recovery of the FAC (yes/no); variables significantly associated; effect of the fracture and outcome.

No Walking Ability Recovery at 6th Month					
*Bivariate Analysis Recovery of the Walking Ability (Yes/No)*				RO Limits	
Events during Admission	χ^2^	*p*	RO	Lower	Upper
Surgical technique by synthesis	10.00	0.002	1.809	1.263	2.589
Start of standing and gait beyond 3rd day PI	4.36	0.037	1.525	1.044	2.227
BI < 90 at discharge	58.85	<0.001	4.549	3.062	6.759
BI < 60 at discharge	11.06	0.001	1.945	1.325	2.856
Bad walking ability at discharge (FAC category 3)	13.99	<0.001	1.999	1.400	2.857
Impairment ≥ 1 category (FAC 1–3) in the ability to walk	8.53	<0.001	1.711	1.205	2.431
Cognitive impairment according to PS ≥ 5	9.99	0.002	1.948	1.301	2.918
Cognitive impairment in at least one category in the PS	5.62	0.018	3.646	1.294	10.273
New institution at discharge	15.82	<0.001	2.708	1.661	4.413
Hemoglobinemia ≤ 8.5 mg/dL	8.46	0.004	1.771	1.217	2.577
Being transfused ≥ 3 packed red blood cells	16.17	0.001	2.572	1.468	4.507
Delirium	21.5	<0.001	2.429	1.675	3.523
Constipation	22.41	<0.001	2.353	1.657	3.342
Impaired renal function during admission	4.56	0.033	1.578	1.057	2.357
Urinary tract infection	8.02	0.005	2.260	1.306	3.913
Acute urine retention	10.09	0.001	2.669	1.470	4.846
New prescription of vitamin D at discharge	13.70	<0.001	1.960	1.382	2.781
Residential destination when discharged from hospital	36.86	<0.001	0.333	0.233	0.474
New institutionalization at discharge	15.82	<0.001	2.708	1.661	4.413

BI: Barthel Index; FAC: Functional Ambulation Classification; RO: Odds Ratio; PI: Post Intervention.

**Table 8:** Binary logistic regression of recovery of the FAC (yes/no); situation during admission.

Income Events and Effects	No Recovery of Baseline Walking Ability at 6th Month					
R^2^ = 0.275	Coef. β	χ^2^ Wald	*p* Value	RO	L. Inf	L. Sup
Age (years)	0.075	18.318	0.000	**1.078**	1.042	1.116
Sex male	0.239	0.937	0.333	1.269	0.783	2.058
Surgical technique synthesis	0.475	4.960	0.026	**1.609**	1.059	2.445
BI at discharge (0–100)	−0.004	0.218	0.641	0.996	0.981	1.012
Walking ability at discharge (FAC 1–3)	−0.183	0.390	0.532	0.832	0.468	1.480
Loss walking ability during admission	0.868	14.706	0.000	**2.382**	1.529	3.712
Loss ≥ 1 category PS during admission	0.219	0.133	0.715	1.245	0.383	4.047
PS (number of errors) at discharge	0.095	2.846	0.092	1.100	0.985	1.229
Residential destination when discharged	−0.433	2.694	0.101	0.648	0.386	1.088
New institutionalization at discharge	0.481	2.150	0.143	1.618	0.851	3.077
Anemia on admission	0.214	0.591	0.442	1.239	0.718	2.137
Be transfused during admission	−0.171	0.435	0.510	0.843	0.506	1.402
Delirium	0.205	0.735	0.391	1.228	0.768	1.964
Constipation	0.266	1.387	0.239	1.304	0.838	2.029
Impaired renal function	−0.075	0.095	0.758	0.928	0.575	1.497
UTI	0.347	1.105	0.293	1.415	0.741	2.701
AUR	0.259	0.526	0.468	1.295	0.644	2.605
New thickeners at hospital discharge	1.673	2.028	0.154	5.328	0.533	53.270
New vitamin D prescription at discharge	0.066	0.088	0.767	1.068	0.692	1.647

BI: Barthel Index; FAC: functional ambulation classification; PS: Pfeiffer Scale; UTI: urinary tract infection; AUR: acute urine retention.

**Table 9:** Profile of patient losing independence in at least one BI category during admission according to binary logistic regression.

	Loss BI ≥ 1 Categories during Admission					
R^2^ = 0.168	β	χ^2^ Wald	*p*	OR	L. Inf	L. Sup
**Age** (years)	0.184	23.588	<0.001	**1.202**	1.116	1.295
Sex: male	−0.151	0.096	0.757	0.860	0.332	2.228
**PS errors number (0–10)**	−0.446	9.799	0.002	**0.640**	0.484	0.847
BI (0–100) at admission	−0.015	0.778	0.378	0.985	0.953	1.018
FAC (1–3) at admission	−0.237	0.299	0.585	0.789	0.338	1.844

BI: Barthel Index; PS: Pfeiffer Scale; FAC: functional ambulation classification.

**Table 10:** Profile of patient losing independence in at least one FAC category (≥2 levels) during admission according to binary logistic regression.

	Change ≥ 1 Category FAC during Admission					
R^2^ = 0.293	β	χ^2^ Wald	*p*	OR	L. Inf	L. Sup
Age (years)	−0.005	0.124	0.725	0.995	0.970	1.021
Sex: male	−0.388	3.145	0.076	0.678	0.441	1.042
**PS errors number (0–10)**	0.129	5.235	0.022	**1.138**	1.019	1.270
BI (0–100) at admission	0.008	0.654	0.419	1.008	0.988	1.028
**FAC (1–3) at admission**	−2.284	53.251	0.000	**0.102**	0.055	0.188

FAC: functional ambulation classification; PS: Pfeiffer Scale; BI: Barthel Index.
